# Uncovering the Dependence of Cascading Failures on Network Topology by Constructing Null Models

**DOI:** 10.3390/e21111119

**Published:** 2019-11-15

**Authors:** Lin Ding, Si-Yuan Liu, Quan Yang, Xiao-Ke Xu

**Affiliations:** 1 School of Computer, University of South China, Hengyang 421001, China; linding@usc.edu.cn (L.D.); yangquan19941024@163.com (Q.Y.); 2 College of Information and Communication Engineering, Dalian Minzu University, Dalian 116600, China; liusiyuan0311@foxmail.com

**Keywords:** complex networks, cascading failures, network topology, null models

## Abstract

Cascading failures are the significant cause of network breakdowns in a variety of complex infrastructure systems. Given such a system, uncovering the dependence of cascading failures on its underlying topology is essential but still not well explored in the field of complex networks. This study offers an original approach to systematically investigate the association between cascading failures and topological variation occurring in realistic complex networks by constructing different types of null models. As an example of its application, we study several standard Internet networks in detail. The null models first transform the original network into a series of randomized networks representing alternate realistic topologies, while taking its basic topological characteristics into account. Then considering the routing rule of shortest-path flow, it is sought to determine the implications of different topological circumstances, and the findings reveal the effects of micro-scale (such as degree distribution, assortativity, and transitivity) and meso-scale (such as rich-club and community structure) features on the cascade damage caused by deliberate node attacks. Our results demonstrate that the proposed method is suitable and promising to comprehensively analyze realistic influence of various topological properties, providing insight into designing the networks to make them more robust against cascading failures.

## 1. Introduction

Complex networks, involving interactive specific nodes abstracted from the real-world systems, have attracted much attention in recent decades [1,2,3]. Many man-made infrastructure systems such as the Internet, transportation networks, and power grids, are examples of complex networks playing essential roles in our modern society. Understanding their robustness concerning random failures and deliberate attacks is of utmost importance and has an increasing interest. Early studies have concentrated on the static failures of a network and the impact of random and deliberate removal of a node (or edge) or group of nodes altogether [4,5,6], while in some cases, the networks undergoing failures may experience a more catastrophic condition as soon as cascading failures take place [7]. For instance, Hub nodes may fail due to targeted attacks. Taking into consideration the inherent dynamics of network flow, the initial removal of only a few nodes may trigger a cascade of overload failures and eventually propagating the failure to a large fraction of the network, leading to a much more devastating result than the case of static failure [8]. Indeed, such cascading failures were found to be particularly relevant for large-scale breakdowns in various infrastructure networks, such as the Internet collapses [9] and huge blackouts in some countries [10]. As these catastrophic incidents can induce excessive losses in a short period, they alarm the whole world and bring serious concern on the dynamics of cascading failure [11,12,13,14].

From the perspective of complex networks, different models were built to imitate cascading phenomena [15,16]. Our point of interest is the type of load models, in which deriving the flow distribution in a network is one of the key issues. Notably Motter et al. built a cascading load model, where node betweenness based on shortest paths is used to represent the flow of physical quantities [8]. Owing to the fact that the shortest-path flow is common in realistic networks such as the Internet and power grids, their model has enjoyed extensive adoption as the basis of various studies [17,18,19,20,21,22,23]. Also, this basic model was extended to consider network information conditions [24], network weights [25], and system laws [26].

Because it is shown that the dynamic behavior of a network largely depends on its topological structure, based on these cascading models, many efforts were made to analyze the topological impact on the cascade robustness of complex networks. Previous methods adopted for the analysis can be divided into two main types: the empirical approach and modeling approach. The two approaches focus on examining the cascade consequences on different real-life networks and traditional model networks respectively, where the preferential attachment model [16,17,18], the small-world model [19,27], and many others [28,29] are examples of such model networks. Although studies such as these have clarified that network topological properties show some relations with the cascade robustness, including degree distribution [18,29], interdependence characteristics [19,20,21], community structure [22,23,24], assortativity [25], and transitivity effects [26,27,28], hardly any attention is paid to enumerate a real-life network robustness to cascading failures in terms of its multi-scale topological features.

As we all know, for a large-scale real-life network, its topological data is generally difficult to be acquired, and once acquired, the network topology is fixed so that it is hard to study the impact of topological variations. Due to the inflexibility of the purely empirical approach, the modeling approach is usually preferable. However, the traditional network modeling can handle only simple microscopic dynamics driving the formation of the network, and thus the resulting networks are universal, which are difficult to approximate full topological properties of a real network. Moreover, when a certain topological parameter of the model network is adjusted to study its influence, accordingly other parameters are often changed simultaneously. Since statistical parameters which define topological properties are not dimensioned, and network size and structure vary widely, the research results from empirical and traditional modeling approaches cannot be carefully compared. Therefore, with the current methods, it is hard to have a sound understanding of the dependence of cascading failures on underlying network topology. So far, for a specific network such as the Internet, the relationship between topological metrics, such as degree distribution, assortativity, transitivity, rich-club, and community structure with respect to the cascading evolution is still unclear. It is thus desirable to develop a novel approach to give the quantitatively accurate evaluation, which helps to take appropriate measures to establish a stronger system.

Recently, null models for real-life networks were increasingly used to analyze structural complexity [30,31], link prediction [32,33], and community detection [34]. In general, a random network, with certain characteristics of a real network, is called a null network of the original one. The null models (networks) may accurately reflect the non-trivial properties of the original network, and can arrange for a precise reference of the original network together with statistical measurements. Therefore, different from the current empirical and modeling approaches, applying null models allow us to comprehensively and explicitly exploit topological features of a real network and systematically study how the modification of these features can affect the cascade robustness. However, to the best knowledge of ours, there is a lack of studies on cascading failures with null models.

In this study, we aim to close this gap by suggesting a novel approach founded on null models to investigate cascading failures on the Internet, and the effect of the cascade with varying topological structures is explored for a given network. To this end, we first construct various null models to generate realistic alternate networks derived from the standard Internet, where different topological properties are considered including degree distribution, assortativity, transitivity, rich-club, and community structure. Considering the distribution of the actual shortest-path flow, the cascading failure propagation triggered by deliberate node attacks is modeled under varying topological conditions of the Internet. For each of network topologies, the size of the largest connected component of the attacked network is monitored. Then the results are used to establish the relationship between the cascading failures and the variations in different topological features. Based on three Internet AS-level networks, our study validates the proposed method and clearly show how micro-scale properties (i.e., degree distribution, assortativity, and transitivity) and meso-scale properties (i.e., rich-club and community structure) exert impacts on the network robustness against cascading failure, where there are substantially different results from those in traditional model networks.

Furthermore, it should be remarked on that both the perception of the cascades with shortest-path flow and null models were deeply studied, but the approach of integration of the two fields is novel and adopted to investigate the relationship between cascading failures and the topological variations occurring in a given realistic network, which is the key contribution of our paper. Although the study is performed in the framework of propagating failures on the Internet, we believe that the proposed approach can be applicable to studying the robustness of other kinds of real-life networks with reasonable modification because the basic models involved are easily extended.

The remainder of the article is arranged as follows. Section 2 introduces topological parameters and null models engaged in generating distinct topological structures of the Internet. Section 3 states the cascading model with the routing rule of shortest-path flow. Section 4 discusses the procedure involved to explore the topological effect on the cascade robustness and tests it on various Internet AS-level networks with results. Finally, Section 5 concludes this work.

## 2. Constructing Null Models of the Internet

The Internet can be represented as a complex graph with *N* nodes and *E* edges, where the nodes can be routers or ASs, and the edges are the physical connections between nodes. The network topology defines how nodes within the network are arranged and connected to each other. A minor shift in the topology, such as edge swapping, can initiate varying the properties of the network that accordingly affect its dynamical behaviors and functions.

### 2.1. Network Parameters

There are many metrics or parameters to describe statistical properties of network topology, but in this study, we restrict ourselves to consider five basic ones: degree distribution, assortativity coefficient, clustering coefficient, rich-club coefficient, and modularity coefficient, which were widely studied in traditional model networks and exported important impacts on a variety of network-based dynamical processes [1,2,3]. The degree distribution p(k) represents the probability with which a node in the network chosen randomly has degree *k*. Scale-free networks widely observed in reality have a power-law degree distribution, namely p(k)∼$k^{-\lambda }$, where λ is the scaling exponent. The specific definitions of other four parameters are as follows:

1. Assortativity Coefficient
  (1) $$\begin{array}{c} r=\frac{E^{-1}\sum_{e_{ij}}k_{i}k_{j}-{\left[E^{-1}\sum_{e_{ij}}\frac{1}{2}\left(k_{i}+k_{j}\right)\right]}^{2}}{E^{-1}\sum_{e_{ij}}\frac{1}{2}\left(k_{i}^{2}+k_{j}^{2}\right)-{\left[E^{-1}\sum_{e_{ij}}\frac{1}{2}\left(k_{i}+k_{j}\right)\right]}^{2}}, \end{array}$$
  where $e_{ij}$ is an edge connecting nodes *i* and *j*; $k_{i}$ and $k_{j}$ denote the degrees of nodes *i* and *j*, respectively.
2. Clustering Coefficient
  (2) $$\begin{array}{c} c=\frac{1}{N}\sum_{i=1}^{N}c_{i}, \end{array}$$
  where the node clustering coefficient $c_{i}=\frac{2\lambda_{i}}{k_{i}\left(k_{i}-1\right)}$, where $\lambda_{i}$ is the number of the edges existing among $k_{i}$ neighbors of node *i*. The clustering coefficient can be used to measure the transitivity property of a real-life network.
3. Rich-club Coefficient
  (3) $$\begin{array}{c} \phi =\frac{M}{n(n-1)/2}=\frac{2M}{n(n-1)}, \end{array}$$
  where *n* and *M* are the numbers of rich nodes and the edges existing among these rich nodes, respectively.
4. Modularity Coefficient
  (4) $$\begin{array}{c} Q=\sum_{i}(h_{ii}-a_{i}^{2}), \end{array}$$
  where $a_{i}$ = $\sum_{w}h_{iw}$ signifies the row (or column) sums, symbolizing the fraction of edges that link to nodes in community *i* and $h_{iw}$ is the fraction of edges in the original network that connect nodes in the subset *i* with nodes in the subset *w*.

### 2.2. Synthetic Networks Generated by Null Models

Given a real network, its topological structure is settled. To investigate the influence of the above topological properties (e.g., degree distribution, assortativity, transitivity, rich-club, and community structure) on cascading dynamics in detail, several null models based on randomized algorithms are considered to generate alternate realistic topologies of the original network. Here, the randomized algorithms cannot just rewire edges of the original network but also randomize some factors on the condition of precisely keeping some original connection properties. It is clear that the topologies thus created can rigorously grasp real topological characteristics as they are derived from the original network.

#### 2.2.1. dK-Series of Null Networks

We consider two approaches with randomized algorithms for constructing null models. One is the dK-series of prospect distributions, where all degree correlations are indicated in d-sized subgraphs of a specified graph [35,36]. This approach can produce null models of different orders, including 0K, 1K, 2K, and 3K that are applied to approach the original network progressively and then spot its micro-scale features at multi-levels. Null networks of all these orders are interconnected, i.e., 0K ⊇ 1K ⊇ 2K ⊇ 3K. Any higher-order null network embraces the features of lower-order null network.

**Figure 1:**
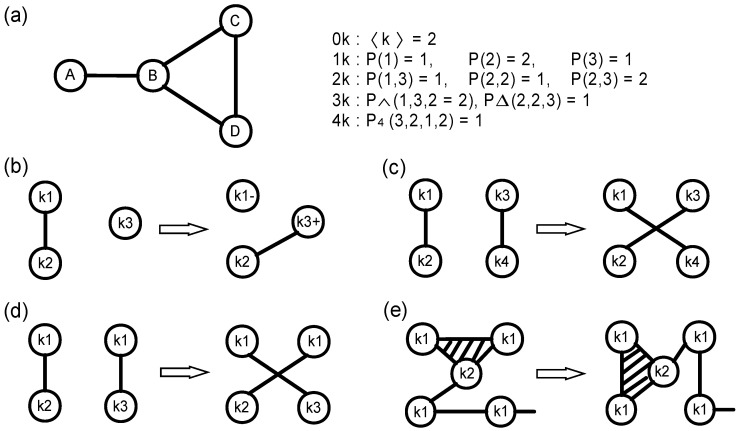
The summary of dK-series null models. (**a**) The properties Pd, d=0,⋯,4, calculated for a given toy network of size 4. The rewiring procedure of (**b**) 0K null network, (**c**) 1K null network, (**d**) 2K null network, and (**e**) 3K null network.

Figure 1a illustrates the process of constructing the properties Pd, which we call the dK-series of null networks. The d=0,⋯, 4 corresponds to different order of dK-series [34]. We use the total number of corresponding subgraphs to represent all the values of *P*. That is to say, P(2,2) = 1 means that the network has one edge between two 2-degree nodes. 0K null network in Figure 1b is the simplest and the most randomized version of the original network, which only retains the number of nodes and the average degree of it. 1K null network maintains the degree distribution of the original network, but it has randomly rewired the link relationship as shown in Figure 1c. 2K null network holds the identical joint degree distribution of the original network in Figure 1d, which means they have the same degree values for the end nodes of each edge. That is to say, they have the same values of assortativity coefficient as the original one. The rewiring procedure of 3K null network is demonstrated in Figure 1e. 3K null and the original networks hold identical clustering coefficient for each node. Thus, with the increase of null model orders (i.e., the increase of the constraints for generating null models), the null networks can be gradually approaching the original network theoretically.

#### 2.2.2. Null Networks of Tunable Properties

Although the above four null models of different orders are useful in understanding the behaviors of the original network, they cannot capture how to control it more efficiently. Therefore, we also consider the approach of the targeted edge-swapping [37], which can create null models with tunable micro-scale properties, such as assortativity, transitivity and meso-scale properties, such as rich-club, community structure. We refer to them as null models with tunable properties.

To obtain the increased and decreased assortativity *r*, we consider null models of increasing and decreasing assortativity respectively, where *r* is tunable [37,38]. Such null networks are constructed as follows. The process of edge swapping is conducted on the original network with preferred *r*. First, two edges $e_{st}$ and $e_{uv}$ are randomly picked up from the network, where the rank of the degrees of nodes *s*, *t*, *u*, and *v* is denoted by $k_{s}$ > $k_{u}$ > $k_{t}$ > $k_{v}$, and $e_{su}$, $e_{tv}$, $e_{sv}$, and $e_{ut}$ do not exist. Then, $e_{st}$ and $e_{uv}$ are removed. For generating the null network of increasing assortativity, we add the edges $e_{su}$ and $e_{tv}$; while for generating the null network of decreasing assortativity, we add the edges $e_{sv}$ and $e_{ut}$. The swapping procedure goes on iteratively until the error between the observed and preferred value is within a very tiny value, such as 0.005.

Similarly, we consider null models of increasing and decreasing transitivity, rich-club property [33,39], and community structure [34], combined with well-controlled parameters *c*, ϕ, and *Q* respectively. Besides, to gain further insights about community structures, we consider null models of rewiring edges within a community and between two communities [34]. The former only varies the inner topology of each community, at the same time maintaining the structural features between communities and the number of communities. The latter only changes the links between two communities but maintains the structural features inside each community.

Note that for the null models of tunable assortativity, when the assortativity coefficient is adjusted, its lower-order property (i.e., the degree distribution) can remain the same as that of the original network. In a similar way, the null models of tunable clustering coefficients can keep its lower-order properties (i.e., the degree distribution and assortativity) unchanged. Moreover, when one meso-scale property, such as the rich-club coefficient or modularity coefficient is adjusted, because the desired value of the property can be obtained only by exchanging a small number of edges in the original network, micro-scale properties of most connectivity between network nodes, including the degree distribution, assortativitity, and transitivity, all can remain almost unchanged. Therefore, the null models in this study allow us to identify how the modification of one meso-scale property affects cascading dynamics in the case of keeping micro-scale properties unchanged in the network.

## 3. Cascading Failure Model

With the desired null networks at hand, these networks are then enriched with data flow. We follow the routing rule of shortest-path flow described in [8]. In the rule, at each time step, one packet is exchanged between every couple of network nodes and transferred along the shortest paths linking them. Under this situation, the load at a node can be denoted by its betweenness [18,19]. This definition method on the load was widely applied to different kinds of realistic networks, including communication networks such as the Internet, transmission systems such as power grids, and transportation networks [17].

The capacity of a node is the highest load that it can handle. Generally, the capacity is restricted by the cost in a real-life network. Therefore, it is sensible to suppose that the capacity $C_{i}$ of node *i* is proportional to its initial load $L_{i}\left(0\right)$ [21]:

(5) $$\begin{array}{c} C_{i}=\left(1+\alpha \right)L_{i}\left(0\right), \end{array}$$

where α(α≥0) is a redundancy parameter. α ≥ 0 guarantees that initially, all nodes work properly (i.e., without overload).

Here, the potential cascading failure is considered to be triggered by removing a single node with the highest load, because many prior studies concerning both model networks and real networks have shown that such a node failure can affect loads at other nodes considerably and thus cause severe loss to the network. Assume such deliberate attack arises at *t* = 1. The removal of the attacked node in general changes the distribution of shortest paths. The traffic load used to go through this node has to be rerouted. Therefore, the loads of some nodes may increase beyond their capacities. Consequently, the corresponding nodes are overloaded and thus fail. Once more nodes fail, the shortest paths among all node pairs and the loads are then recalculated based on the topological modification. The process of node cascading failure and the load redistribution is iterated until no node fails, at which point the cascading propagation course is considered as being accomplished.

The dynamical function of a real-life network depends on the node capability to communicate efficiently with each other. Suppose the number of nodes in the largest component before and after the cascade to be *N* and $N^{\prime }$ respectively, without loss of generality, the damage triggered by a cascade is calculated by the relative size *G* of the largest connected component, i.e., G=N′N′NN. As *G* of the attacked networks is checked, the profile of this parameter variation can display the network invulnerability and robustness against cascading failure. Obviously, the greater the value of the index *G*, the better the network robustness against cascading failure.

## 4. Main Results

This section adopts real network data to identify the topological impact on the cascade damage in detail. We consider three Internet AS-level networks [40]. They contains 3015 nodes, 530 nodes and 493 nodes, respectively. The data were collected from online data and reports of the University of Oregon Route Views Project. For each of the original networks, their multiple alternate networks are generated by employing four null models of different orders as well as ten null models with tunable properties, and the cascading model is applied to them. Extensive simulations are implemented to reveal the potential relationship between the cascade robustness with topological variations occurring in the Internet network. In the simulations, each curve for null networks is averaged over 30 realizations. It should be noted that although the following analysis is performed by using the real Internet networks, our framework and proposed approach involved are applicable to other kinds of realistic networks.

### 4.1. Cascading Failures in Null Networks of Different Orders

**Figure 2:**
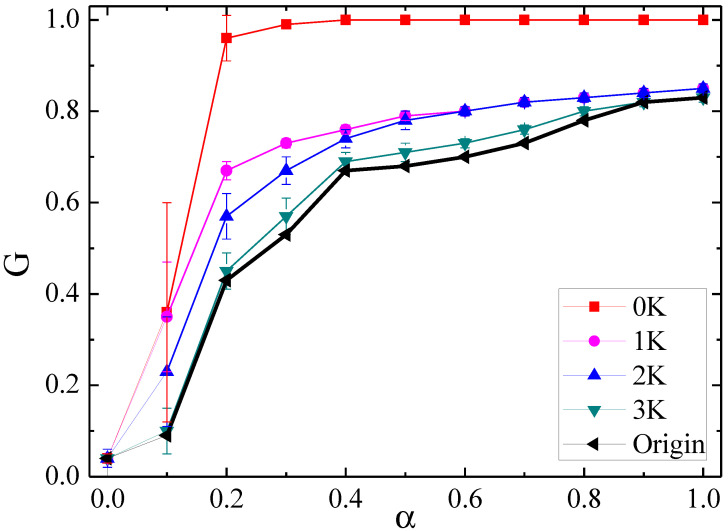
The relationship between *G* and α in null networks of different orders for the Internet with 3015 nodes and 5156 edges.

Let us first investigate cascading failures in null networks of different orders (0K–3K). Figure 2 compares the results of cascading failures in these null networks to that in the original Internet with 3015 nodes. The curves demonstrate the association between the relative size *G* and the redundancy parameter α under topological variation. As expecting in Figure 2, *G* monotonically increases with the increase of α for each curve. Based on the definition of the cascading model, increasing α means each node has more capacity redundancy to receive the redistributed load from failed nodes, which will reduce the likelihood of subsequent overload failures. Then the robustness of the whole network becomes stronger (i.e., the robustness measurement index *G* increases). Moreover, we observe that the network robustness against cascading failure is gradually weakened with increasing the order of null networks from 0K to 3K, leading to getting closer to the original one. This can be explained by the different role of each topological feature in the network robustness.

Table 1 shows the variation of topological parameters for the Internet with 3015 nodes and its corresponding 0K–3K null networks. As it is seen, 0k null networks, which are obtained after sufficient randomization on the original network, only have the same number *N* of nodes and average degree <*k*> as the origin. Moreover, they display a Poisson degree distribution rather than a power-law (scale-free) distribution which the original network shows. Such homogeneous structure makes them much more robust than the original heterogeneous network. This is in line with prior studies on cascading failures in traditional model networks [8,23,29], where cascading failure was shown to occur less likely in a homogeneous network than in a heterogeneous one. Because it was shown that cascading dynamics are strongly related to the degree distribution of a network, it also raises a new question whether it is certain that both of them have the similar ability in resisting cascading failures if a network rigorously grasps the degree distribution of the original network.

In this work, 1K null networks are such examples but clearly perform very different ability from the original network. Compared with 1K null networks, the robustness of 2K null networks is further worse, and then their curve is closer to that of the original network, because 2K null networks maintain more micro-scale structures (i.e., assortativity). Though, there is yet a great difference between 2K null networks and the original one. Furthermore, the evolution of 3K null networks more resembles that of the original network, because they can preserve the transitivity characteristics of the original network. The above findings, hence, confirm that different topological features of networks all have great impact on the outcomes of cascading dynamics taking place on them. Actually, most previous studies based on network models pay attention to the degree distribution to evaluate the robustness of a network against cascading failures [23,29]. This study clearly shows that the degree distribution is not enough to guarantee the robustness of a real-life network, and 2K and 3K micro-scale structures (i.e., assortativity and transitivity) are also significant properties. Therefore, further studies are needed to reveal how the modification of assortativity, transitivity and higher-order properties affects cascading failures in case of keeping its lower-order properties of the original network.

### 4.2. Cascading Failures in Null Networks of Tunable Assortativity

**Figure 3:**
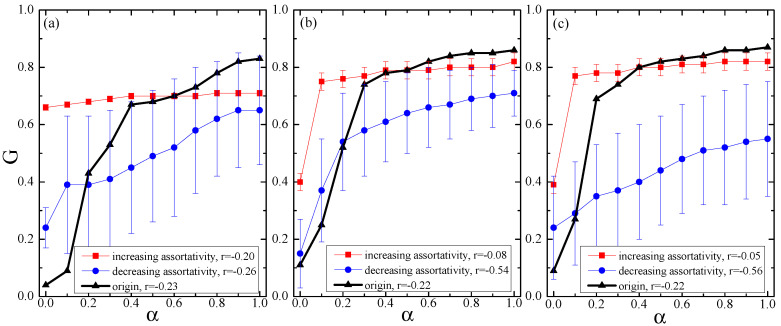
The relationship between *G* and α in null networks of (**a**) increasing assortativity (*r* = −0.20) and decreasing assortativity (*r* = −0.26) for the Internet with 3015 nodes and 5156 edges (*r* = −0.23), (**b**) increasing assortativity (*r* = −0.08) and decreasing assortativity (*r* = −0.54) for the Internet with 530 nodes and 1289 edges (*r* = −0.22), and (**c**) increasing assortativity (*r* = −0.05) and decreasing assortativity (*r* = −0.56) for the Internet with 493 nodes and 1234 edges (*r* = −0.22).

Figure 3a shows the relationship between *G* and α in null networks of increasing assortativity (*r* = −0.20) and decreasing assortativity (*r* = −0.26) for the Internet with 3015 nodes, where the *r* value of the original network is −0.23. In contrast to the case of *r* = −0.26, for the curve of *r* = −0.20, the values of the error bars are so small(about 0.01) that we cannot see them. This can be explained by the fact that under the algorithm of the edge-swapping of increasing assortativity, the difference of connectivity patterns of all generated networks is small due to the constraint of a few nodes with high degree in the original network. Meanwhile, the structure (i.e., a large number of nodes with low degree) of the network can make all generated null networks of decreasing assortativity show a relatively large difference between their connectivity patterns. The difference of connectivity patterns accordingly affect the difference of cascade results, leading to the difference of the error bars of the two curves. In addition, from the curve of *r* = −0.20, we can see that α exerts a relatively small impact on the cascade results, which is different from the case of the other two curves. The reason is also related to the network structure.

More importantly, Figure 3a illustrates that *r* has a strong effect on the network robustness. The networks of increasing assortativity take more robustness and resistance to the damage of the cascade as compared to those of decreasing assortativity for any choice of parameter α. However, compared with the origin, there exist crossover points $\alpha_{c}$ ($\alpha_{c}$ ≈ 0.6) for the networks of increasing assortativity and $\alpha_{s}$ ($\alpha_{s}$ ≈ 0.2) for the networks of decreasing assortativity. When $\alpha_{s}$ < α < $\alpha_{c}$, the cascade robustness of the network with higher assortativity indeed increases. In contrast, when α > $\alpha_{c}$ or α < $\alpha_{s}$, the network with lower assortativity performs better. This means that the network robustness does not always increase monotonically with its assortativity, which is sensitive to the capacity redundancy. Similar evolution with different cross points can be observed in Figure 3b,c. It can also further confirm that the capacity redundancy is considered to determine how the assortativity affects the network robustness. This is different from the results obtained in traditional model networks [25], which indicate that the robustness of complex networks against cascading failures varied monotonously with the variations of its assortativity regardless of the capacity redundancy.

### 4.3. Cascading Failures in Null Networks of Tunable Clustering Coefficient

**Figure 4:**
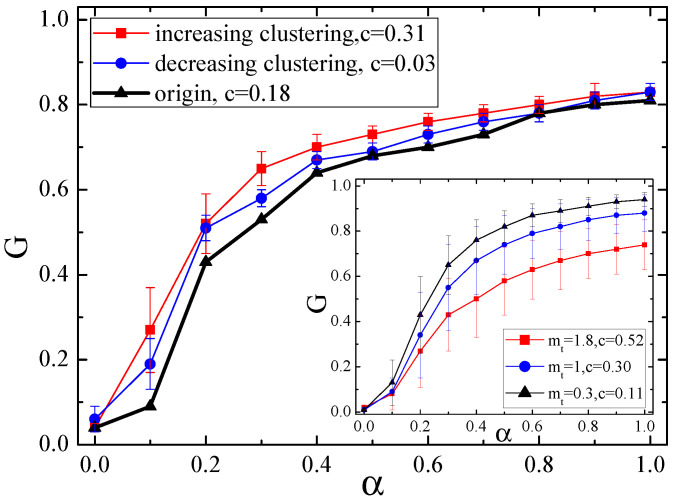
The relationship between *G* and α in null networks of increasing transitivity (*c* = 0.31) and decreasing transitivity (*c* = 0.03) for the Internet. For comparison, the inset shows the relationship between *G* and α in traditional networks (i.e., HK scale-free networks) with different values of clustering parameter $m_{t}$. Each data is averaged over 50 individual runs.

Figure 4 plots the relationship between *G* and α in null networks of increasing transitivity (*c* = 0.31) and decreasing transitivity (*c* = 0.03) for the Internet with 3015 nodes, where the *c* value of the original network is 0.18. It can be seen that the networks of both increasing transitivity and decreasing transitivity are more invulnerable to cascading failure as compared to the original network, and the impact of increasing transitivity on promoting the network robustness is more prominent. For example, in the case of α = 0.3, increasing transitivity and decreasing transitivity make the value of *G* increase from 0.53 to 0.65 and 0.58, respectively.

Such results also indicate a non-monotonic behavior between the transitivity property and the cascade robustness. This phenomenon is different from the result obtained in those traditional model networks as shown in the subgraph of Figure 4. Here we use a typical model, namely the HK scale-free network model proposed by Holm and Kim [26], in which the clustering coefficient can be adjusted by a particular control factor $m_{t}$. In the case of $m_{t}$ = 0, the HK model degenerates into the well-known Barabási-Albert(BA) model [41]. The larger the $m_{t}$ value, the larger the clustering coefficient *c*. When applying our cascading model to the scale-free networks with tunable clustering coefficient *c*, the subgraph of Figure 4 shows that the robustness of such networks is monotonously reduced with the increase of *c*. Obviously, this is not in accordance with the result of our proposed null networks. Coupled with the results in Figure 3, we can confirm that due to the structural complexity of real-life networks, from which deviations of traditional model networks can bring great impacts on understanding and controlling cascading behaviors, and hence constructing null models for empirical analysis of cascading failures is of great significance.

### 4.4. Cascading Failures in Null Networks of Tunable Rich-Club Property

Until now, we have shown the effects of micro-scale features (i.e., assortativity and transitivity) on the cascade. However, we do not know what the effects of meso-scale features are on that. The rich-club and community structure are two typical meso-scale features of the Internet. In the following, the impact of them each on cascading behaviors will be also investigated.

**Figure 5:**
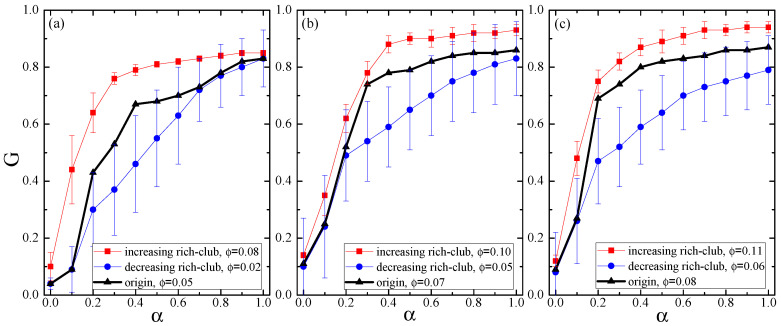
The relationship between *G* and α in null networks of (**a**) increasing rich-club (ϕ = 0.08) and decreasing rich-club (ϕ = 0.02) for the Internet with 3015 nodes and 5156 edges (ϕ = 0.05), (**b**) increasing rich-club (ϕ = 0.10) and decreasing rich-club (ϕ = 0.05) for the Internet with 530 nodes and 1289 edges (ϕ = 0.07), and (**c**) increasing rich-club (ϕ = 0.11) and decreasing rich-club (ϕ = 0.06) for the Internet with 493 nodes and 1234 edges (ϕ = 0.08).

Figure 5a shows the relationship between *G* and α in null networks of increasing rich-club property (ϕ = 0.08) and decreasing rich-club property (ϕ = 0.02) for the Internet with 3015 nodes, where the ϕ value of the original network is 0.05. Clearly, one can see that the ϕ value is positively related to the network invulnerability against cascading failures, i.e., the higher ϕ, the more desirable network behavior in resisting cascading failures. The similar evolutions can also be observed in Figure 5b,c. This suggests a plausible way to enhance network robustness by increasing rich-club property.

### 4.5. Cascading Failures in Null Networks of Tunable Community Structure

**Figure 6:**
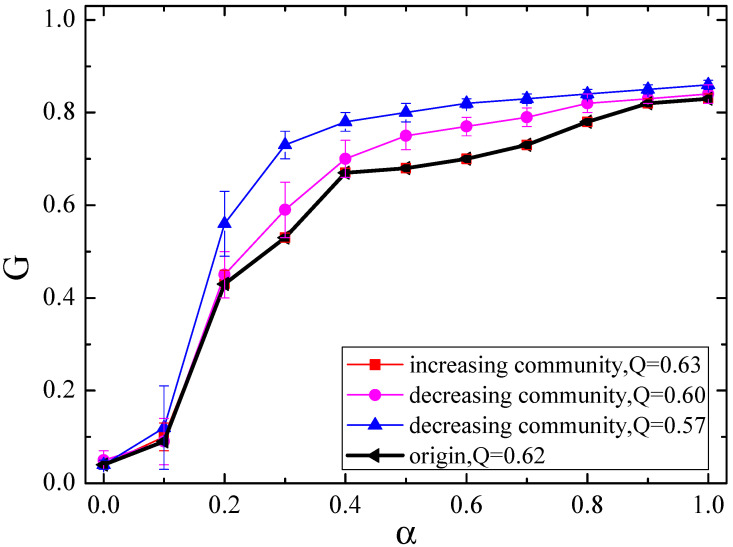
The relationship between *G* and α in null networks of increasing community structure (*Q* = 0.63) and decreasing community structure (*Q* = 0.60,0.57, respectively) for the Internet.

In Figure 6, we plot the relationship between *G* and α in null networks of increasing community structure (*Q* = 0.63) and decreasing community structure (*Q* = 0.60,0.57, respectively) for the Internet with 3015 nodes, where the original *Q* value is 0.62. With decreasing *Q* of the original network, the cascade robustness of the null networks becomes stronger. However, when we increase *Q*, the robustness is almost the same as that of the original one. This can be explained by the fact that the original version of the Internet has already a clear community characteristic. Using the algorithm of increasing community structure, the *Q* value can be adjusted to the maximum value (*Q* = 0.63), which is only increased by 0.01 as compared to the original value. Such enhancement of the community structure has no obvious effect on the robustness of the original network.

**Figure 7:**
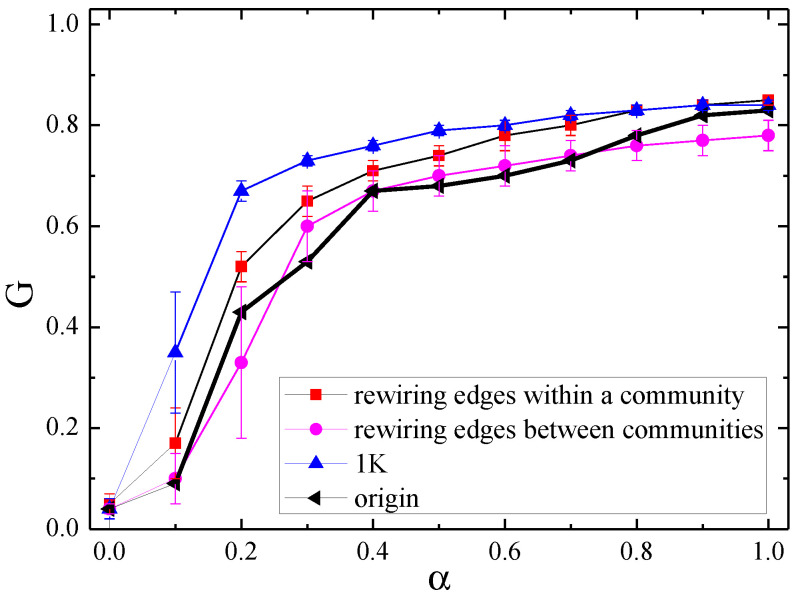
The relationship between *G* and α in null networks of edge swapping within a community and between communities for the Internet.

The characteristic of community structure is that the density of inner edges among communities is relatively greater than the density of external edges. To disclose the community structure effect in detail, Figure 7 displays the relationship between *G* and α in null networks of rewiring edges within a community and between communities for the Internet with 3015 nodes. For generating these two null networks, we consider the edge-swapping algorithm of the 1K null model to only destroy the inward structure of each community and the external structure of all communities, respectively. It should be noticed that both of them are different from the classical 1K null network, which is based on rewiring edges within the whole original network and thus destroys the meso-scale characteristics completely.

As seen in Figure 7, different kinds of edges play different roles in the effects of cascading failure. Rewiring edges inside a community (i.e., the random exchange of inner edges) makes the network stronger to resist cascading failures, yet for rewiring edges among communities (i.e., the random exchange of exterior edges), the effect on promoting the network invulnerability is not clear enough because of their fluctuations. Furthermore, the network with the modification of inner edges is still more vulnerable than the 1K null network. Because the 1K null network completely destroys community structures of the original network, its modularity is lower than the other three cases, and the smaller modularity is in favor of the cascade robustness.

## 5. Conclusions

In this work, Internet networks were researched in terms of topology and the association with the cascade robustness was established. Considering realistic topological characteristics including degree distribution, assortativity, transitivity, rich-club coefficient, and modularity, we generated multiple null networks derived from three Internet AS-level networks. The methods used for the generation of null networks offer feasible network configurations inherently. By considering the routing rule of shortest-path flow, the effect of cascade-based attacks was investigated in each of the original networks and its various null networks with the topological variation.

By comparing the largest connected sizes of these attacked networks, our results reveal that the degree distribution is not enough to identify the robustness of a network, and micro-scale structures (i.e., assortativity and transitivity) and meso-scale structures (i.e., rich-club and community structure) are also important for that. In detail, at the micro-scale level, both assortativity and clustering coefficient show a non-monotonic behavior with network robustness. Moreover, the impact of the assortativity on network robustness is related to the capacity redundancy of nodes. In comparison to decreasing transitivity, increasing transitivity contributes more to the promotion of network robustness. At the meso-scale level, the rich-club structure is positively related to network robustness, indicating that increasing it leads to stronger robustness. In contrast, the modularity is inversely related to network robustness, indicating that the network robustness increases with reducing modularity characteristic. In addition, the topological structure within every community plays a more significant role in improving the robustness as compared to that between communities.

The results obtained here are meaningful in guiding the construction or optimization of the Internet to resist the propagation of cascading failure effectively. Inspired by this work, we will aim to understand the relationship between complex characteristics of the Internet and cascading failure more comprehensively in our future studies. For example, an interesting challenge is to analyze interdependence of the systems in such a way to launch a robust network design. Our results also demonstrate the significance of constructing null networks for understanding and analysis of cascading failure in more real-world networks, because it can produce substantially different results from those in traditional model networks. We expect the analysis method proposed here is promising for extended applications in studying robust systems with different network structures in the real-world other than the Internet as well. 

## Figures and Tables

**Table 1:** The variation of topological parameters for the Internet with 3015 nodes and its corresponding 0K–3K null networks. Each data point for 0K–3K null networks is averaged over 30 different network realizations.

Network	*N*	<*k*>	*p*(*k*)	*γ*	*r*	*c*
Origin	3015	3.42	Power-law	2.5	−0.23	0.18
0K	3015	3.42	Poisson	−	−0.008	0.0009
1K	3015	3.42	Power-law	2.5	−0.22	0.10
2K	3015	3.42	Power-law	2.5	−0.23	0.12
3K	3015	3.42	Power-law	2.5	−0.23	0.18
